# “They Are Not Going to Be Happy”: An Ethnographic Study of the Prioritization of Patients Awaiting Elective Surgery in an Academic Hospital in the Netherlands

**DOI:** 10.1177/0272989X261422220

**Published:** 2026-02-21

**Authors:** Philipa Mos, Bert de Graaff, Michelle Heijke, Hester Lingsma, Robert Jan Baatenburg de Jong, Vivian Reckers-Droog

**Affiliations:** Department of Health Law & Ethics, Erasmus University Rotterdam, Erasmus School of Health Policy & Management, Rotterdam, the Netherlands; Department of Health Care Governance, Erasmus University Rotterdam, Erasmus School of Health Policy & Management, Rotterdam, the Netherlands; Department of Public Health, Erasmus University Medical Centre, Rotterdam, the Netherlands; Department of Public Health, Erasmus University Medical Centre, Rotterdam, the Netherlands; Department of Otorhinolaryngology and Head & Neck Surgery, Erasmus University Medical Centre, Rotterdam, the Netherlands; Department of Health Economics, Erasmus University Rotterdam, Erasmus School of Health Policy & Management, Rotterdam, the Netherlands

**Keywords:** decision support techniques, elective surgical procedures, professional autonomy, waiting lists

## Abstract

**Background:**

To reduce variation in waiting time for elective surgery, a Dutch academic hospital introduced a classification system based on urgency scores to standardize decision making. Physicians, however, retain clinical discretion in assigning urgency scores. This facilitates the provision of personalized and efficient care but may also create variation between patients and lack of transparency. The aim of this study was to describe the prioritization of patients awaiting elective surgery, including the use of urgency scores, and to explore explanations for discrepancies between assigned scores and actual waiting times.

**Methods:**

We conducted an ethnographic study combining interviews with physicians and observations of elective surgery planners in the academic hospital. Data were analyzed thematically, guided by 3 sensitizing concepts: professional autonomy, emotions, and traditions.

**Results:**

The prioritization of patients awaiting elective surgery begins with physicians’ assessment of urgency and concludes with planners drafting the schedule. The assessment is guided by clinical parameters, patient- and physician-related factors, and logistical constraints. Importantly, the prioritization of patients for elective surgery is shaped by subjective and affective considerations, customary decision-making practices, as well as the considerable professional autonomy of physicians and planners.

**Conclusions:**

Standardized prioritization tools, such as urgency scores, may reduce unjustified variation in waiting times, but initial resistance to their implementation can hamper their use in decision-making practice. Moreover, such tools alone may fail to capture the complexity of clinical practice and the importance of the expertise and experience of physicians and planners therein. Rather than relying solely on stricter adherence to urgency scores, prioritization processes may be strengthened by facilitating communication and feedback exchanges to support a more integrated and context-specific approach that considers the complexity of clinical practice.

**Highlights:**

- Standardized decision-making tools are implemented to standardize and support the prioritization of patients awaiting elective surgery.
- Prioritization decisions are made by different professionals, and nonclinical factors that include subjective perceptions and logistic constraints may guide these decisions.
- Standardized tools inadequately capture the complexity of clinical decision making and the professional autonomy physicians and planners.

## Introduction

In the face of unlimited demand for health care, financial constraints, and staffing and capacity shortages, decisions regarding the allocation of health care resources are inevitable.^1,2^ Such decisions can be made explicitly on the national level, based on transparent criteria,^3,4^ or implicitly at the bedside by health professionals on a patient-by-patient basis.^1,5^ Considerable professional autonomy allows health professionals to address the unique needs of individual patients.^6–8^ However, obtaining insight into “the secret garden of clinical rationing”^6^ is complicated. Evidence suggests that decisions made at the bedside level may be guided by patients’ preferences, expectations, health literacy, the physician–patient relationship, physicians’ experience, traditions, cognitive biases, and heuristics.^6,9–11^ As a result, decisions may, all else being equal, not always be consistent, and variation in care provision and waiting times exists that cannot be explained by clinical factors.^6,12,13^

In the Netherlands, variation in the waiting times for emergency surgery prompted the development of a national guideline for prioritizing patients based on clinical urgency.^14^ Yet, no national guideline exists to reduce variation in waiting time for elective surgery (i.e., surgery that can be planned ahead, with timelines ranging from 48 h up to 6 mo). Therefore, an academic hospital in the Netherlands implemented a classification system based on urgency scores to standardize decision making by physicians. Physicians assign a clinical urgency score (A–K) to each patient, corresponding to a recommended time frame for surgery. An urgency score A indicates the highest clinical urgency and shortest time frame, while K reflects the lowest clinical urgency and a longer time frame. Patients’ surgeries are then scheduled based on their urgency scores by a designated planner, typically an administrative staff member without formal medical training but experienced in scheduling through practice.

While the introduction of urgency scores was meant to standardize decision making by physicians, they retain discretion in the prioritization of patients, and internal hospital data indicated that actual waiting times do not always correspond with assigned urgency scores, suggesting that variation persists. Currently, insight into the process of assigning urgency scores and the prioritization of patients on the waiting list for elective surgery is lacking. This study aims to provide insight into the prioritization of patients awaiting elective surgery in an academic hospital in the Netherlands, including the use of urgency scores, and to explore explanations for discrepancies between assigned scores and actual waiting times.

Academic hospitals play a key role in setting standards for clinical practice and often serve as frontrunners in innovation.^15^ Therefore, the results of this study are relevant not only for the broader health care system in the Netherlands but also for other countries where bedside-level decisions determine access to surgery and where efforts focus on reducing variation.

## Methods

### Study Design and Participants

We conducted an explorative ethnographic study combining 4 semi-structured interviews with 6 physicians and 5 nonparticipatory observations of 8 planners across 4 surgical departments in an academic hospital in the Netherlands. This design was chosen as it allows for an in-depth examination of social interactions, decision-making processes, and contextual factors that may influence prioritization.^16,17^ Three of the 4 surgical departments were selected based on an explorative statistical analysis of internal data, which indicated that, after correcting for assigned urgency scores, the median actual waiting times in these departments statistically significantly exceeded the planned waiting times for elective surgery. Note that we do not provide additional information on this to safeguard the anonymity of the setting and participants involved. These 3 departments had close operational ties with a fourth department, which was therefore also selected. From each department, we recruited physicians for the interviews, and we used snowball sampling to recruit planners for the observations.

We obtained written informed consent from interview participants. For the observations, we obtained written consent from the primary participant and verbal consent from others present. Participants did not receive any compensation for their contribution to this study. Ethical approval for this study was obtained from the Ethical Review Committee of the Erasmus School of Health Policy & Management (ETH2425-0303).

### Data Collection

The interviews and observations were conducted by the first author. For the semi-structured interviews, a topic list (Supplementary Materials S1) was used. The list covered participants’ role in the assignment of urgency scores, the prioritization of patients awaiting elective surgery, the communication between physicians and planners, and perceived (dis)advantages of the decision-making process. The interviews were audio-recorded and transcribed verbatim using Amberscript. Observations lasted for the duration of a morning or an afternoon, resulting in a total of 18 h of observation. All observations were nonparticipatory, and participants were asked to continue their regular work during the observation. When appropriate, the participants were asked to briefly explain what they were doing and why. The observations focused primarily on one planner, although other planners were occasionally present. In those instances, the focus was broadened to also include them to document collaboration and any differences in routines. During each observation, written field notes were taken, documenting details such as reactions to patients, communication with colleagues, and the handling of emergencies. Field notes were used to write a detailed report after the observation.

### Data Analysis

We thematically analyzed the interview transcripts and observation reports using a semi-inductive approach. Following data collection and before the analysis, we identified 3 sensitizing concepts to guide our analysis: the role of professional autonomy, emotions, and traditions in decision making.^18^ We developed the thematic framework in 4 steps. First, the first author imported the transcripts and reports into ATLAS.ti25. Second, the first author drafted a preliminary framework comprising themes and categories describing the prioritization of patients awaiting elective surgery based on an initial round of coding of all data. Third, the third and final author independently coded a random selection of 50% of the data to ensure that the themes and categories comprehensively reflected the data. Through collaborative discussion, the authors further developed the thematic framework. Finally, the first author performed a second round of coding of all data to refine that framework.

We report our findings following the Standards for Reporting Qualitative Research (SRQR) guideline.^19,20^

## Results

From the thematic analysis, 4 main themes emerged: 1) decision-making processes, 2) professional autonomy and expertise, 3) affective dimensions of decision making, and 4) professional habits and customs. Below follows the narrative summary of these themes, supported by interview quotes and excerpts from the observation reports. Table 1 provides an overview of the participants, including their role(s) and departmental affiliations.

**Table 1:** Overview of participants.

ID No.	Role	Department
1	Physician	A
2	Physician	A
3	Physician–planner	B
4	Physician–planner	B
5	Physician	C
6	Physician	D
7	Planner	A
8	Planner	D
9	Planner	D
10	Planner	D
11	Planner	C
12	Planner	C

### Decision-Making Processes

**Figure 1:**
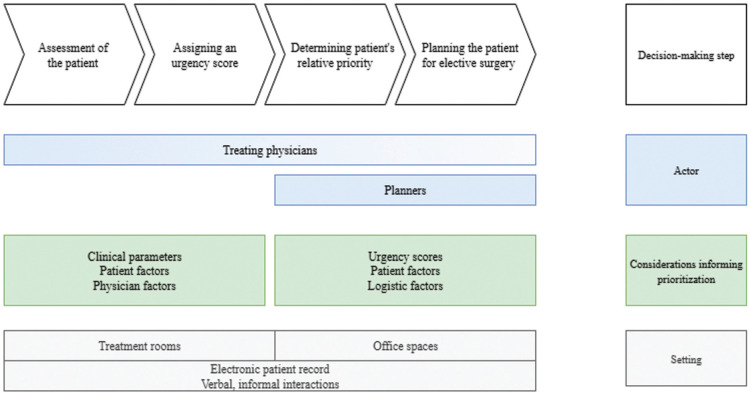
Overview of the decision-making process of prioritization of patients awaiting elective surgery.

Figure 1 provides a graphical overview of how patients awaiting elective surgery are prioritized using urgency scores, highlighting the 4 stages of the process, relevant actors, considerations guiding them, and setting.

The decision-making process typically starts with physicians assigning an urgency score to a patient. According to participants, urgency scores reflect clinical priority in terms of a maximum acceptable waiting time. Physicians explained that the assignment is guided by the patient’s primary disease, general health status, pain, the growth rate of a malignancy (for oncology patients), and neurologic deficits when relevant. For oncology patients specifically, the standard of care is to treat patients within 6 wk of diagnosis. However, physicians treating oncology patients noted they sometimes deviate from this guideline based on their clinical judgment. As one physician put it, the assignment of urgency scores is “not cast in concrete” (ID2, physician). Patients are usually informed about the expected time frame for their surgery but not about the urgency score assigned to them. After a physician manually enters the score into the electronic patient record (EPD), the patient is placed on the waiting list. The clinical ward where physicians enter the scores and the office where planners schedule surgeries are separated, and urgency scores are used to digitally communicate patients’ clinical urgency from physicians to planners.

Planners continuously and iteratively determine the relative priority of patients on the waiting list to draft the surgical schedule. Planners have insight into patients’ urgency scores, the admission deadline, the application date, and the expected duration of the surgical procedure, and they can manually adjust the sorting of the waiting list based on this information. While urgency scores indicate the ranking of patients based on clinical priority, which patient is eventually selected for surgery first is primarily guided by logistical constraints, particularly the availability of postoperative beds and the expected duration of the procedure. Longer surgeries are more difficult to schedule, and consequently, these patients tend to remain on the waiting list for a longer period even when they have high urgency scores. When the admission deadline is exceeded, planners consult the treating physician to ensure waiting longer is medically safe. One physician noted: “Anyone who does not have to be treated within 6 weeks can wait another week or 2, and someone with an admission deadline of 3 months can wait another month” (ID4, physician–planner). When drafting the schedule, planners typically review the waiting list for procedures that fit the available time slot, sometimes prioritizing patients lower on the waiting list:

The planner adjusts the sorting of the waiting list [on their computer]. First, based on urgency score, from most to least urgent, then by application date, in chronological order. The planner scans the waiting list top-down to select a patient for an operating room time of 60 minutes. The planner selects a patient somewhat in the middle of the list while another patient was listed above. (Observation 1, 18-02-2025).

Planning timelines vary by department, depending on the average level of urgency of their patients. Because elective surgeries are performed in the same operating rooms (ORs) as emergency procedures, emergencies may disrupt the schedule for elective surgeries. These interruptions, as well as other disruptions such as unexpected OR closures, a surgery exceeding its expected duration, or planning errors, necessitate rescheduling. Planners then decide whose surgeries to postpone based on urgency scores, the number of times a patient’s surgery has previously been canceled due to schedule disruptions, the duration, and whether the procedure is mono- or multidisciplinary. The relative weight of these considerations may vary. A planner explained: “It matters how many times a patient has been canceled for surgery; someone whose surgery has been canceled several times is given priority over another patient, even if the latter’s urgency is higher” (ID7, planner). Furthermore, multidisciplinary surgeries or those with long durations are preferably not postponed, as these are harder to reschedule on short notice.

An emergency patient is admitted to the hospital, needing surgery within 48 hours. Although already packed, the emergency patient is added to the next day’s planning. Meanwhile, the planner is busy arranging a postop bed for the emergency patient. Another emergency patient comes in, requiring surgery within 24 hours. Two scheduled patients must be postponed. One of them had already been rescheduled once and the planner promises the patient to reschedule its surgery on short notice. Minutes later, another emergency patient is reported. The first emergency patient is pushed to the next day, despite the risk of nerve damage, and the schedule needs revision again. The patient who was just assured to be scheduled for surgery in a few days is postponed again. (Observation 2, 13-03-2025)

### Professional Autonomy and Expertise

Both physicians and planners exercise professional autonomy in prioritization decisions, derived from their distinct expertise. Physicians retain sole discretion in assigning the urgency score, a clinical judgment that is not defined in national or hospital guidelines and is not subject to consultation within the department. Thus, within the classification system featuring urgency scores, physicians exercise considerable professional autonomy in assessing clinical urgency and determining the corresponding score. Physicians emphasize the importance of this autonomy, arguing it allows them to account for differences between patients, as opposed to urgency scores. One physician noted: “We assign an urgency score, but we are limited in terms of these codes, they almost never really fit. Usually, I just select 6 weeks” (ID3, physician–planner).

According to physicians, planners do not necessarily have the medical expertise required for assessing urgency. One physician noted: “In principle, we are the only ones who, medically speaking, can indicate the ranking” (ID3, physician–planner). While this suggests physicians may believe that prioritization decisions rely solely on their judgment, other factors also influence these decisions, which only planners have oversight of. Through experience, planners acquire expert skills in coordinating the various tasks associated with drafting the surgical schedule—including communicating with patients, managing physicians’ calendars, ordering protheses, and arranging postoperative beds. One physician appreciated planners’ expertise: “they are outstanding at what they do” (ID5, physician). Moreover, based on the observations, planning assistants conduct their tasks with minimal oversight from physicians, reflecting trust in their judgments and experience, as well as a considerable level of autonomy.

Nonetheless, interactions between physicians and planners reveal a hierarchy based on clinical expertise. While planners possess the expertise and oversight necessary to draft the surgical schedule, physicians may intervene and overrule their decisions when they feel their patient should be “on the table” (ID5, physician) as soon as possible. This hierarchy between physicians and planners is stressed by a planner expressing, “I am not medically trained, so I trust what the physician tells me” (ID7, planner).

### Affective Aspects of Decision Making

Prioritization decisions are informed by clinical urgency, limited by logistical constraints, and influenced by perceptions of patients formed through interactions between them and physicians and planners. These perceptions seem to evoke either sympathy or apathy toward individual patients, potentially influencing the urgency score and subsequent planning. In some cases, physicians appear to incorporate the patient’s preferences when they assign the urgency score. One physician noted: “It does happen that one thinks: I want to help this patient; I assign a higher urgency score” (ID5, physician). Another physician confirmed this by referencing the practices of colleagues: “If a colleague believes a certain patient needs to be admitted within 3 weeks, then they will adjust the urgency accordingly. But the reasoning behind it is not always that clear” (ID3, physician–planner). However, the physician did not make a habit of challenging the clinical decisions of colleagues.

A planner calls the physician to ask whether the surgery of a specific patient can be rescheduled to the following week. Talking on speaker phone, the physician immediately recalls the patient, who “keeps calling everyone all the time.” Therefore, they had indicated a high urgency. Later, the planner notes that some patients are indeed “frequent callers” and—although disapproving of this behavior—they often schedule such patients sooner to be done with them. (Observation 5, 21-03-2025)

Planners are the primary point of contact for patients on the waiting list and therefore frequently interact with patients. Patients communicate their symptoms, concerns, preferences, or frustrations about waiting times to them, based on which planners form perceptions of patients that can influence the planning in positive and negative ways. Planners have vivid memories of some patients and earlier interactions with them, particularly those who frequently call or whose surgeries have been rescheduled. Planners at times referred to patients affectionately: “that poor soul is totally out of sorts” (ID10, planner) and “this is my buddy” (ID9, planner). Patients who are flexible are viewed more positively, in contrast to those unavailable due to work or holidays. These factors may affect how urgent a planner perceives the surgery is, particularly for the patient, and the effort they put into rescheduling it in case it is cancelled.

When scanning the waiting list, the planner hums and nods in recognition. When opening the record of the first patient on the list, notes about the patient appear. The patient was scheduled for surgery 2 times but cancelled, with a pretext. Aloud, the planner says: “I am seriously considering labeling the patient as not plannable and removing them from the waiting list.” (Observation 1, 18-02-2025)

Although planners expressed caution of prioritizing patients based on such perceptions, they acknowledge that these may affect their decisions in practice. Based on the interviews and observations, decisions guided by affective considerations are later rationalized using formal or clinical decision criteria.

A patient on the waiting list calls, expressing pain, judging from the planner’s response. Initially, the planner responds that, “without wanting to be unkind,” pain does not expedite the planning. The planner glances at the waiting list and the EPD. The patient is third on the waiting list. Then, the planner reviews the schedule, sees an opening, and schedules the patient for surgery, subsequently informing the patient. In the memo in the EPD, the planner documents the date the patient called and indicated being in a lot of pain. (Observation 3, 17-03-2025)

The emotional burden related to being unable to grant all patients access also influences the prioritization decisions of physicians as well as job satisfaction. One physician described rescheduling a surgery as being “awful, something I can’t just leave behind at the hospital when I go home” (ID3, physician–planner). For planners, this burden appears related to having to disappoint patients. They note that physicians sometimes raise unrealistic expectations among patients about expected waiting times. They believe this encourages patients to call planners more frequently, resulting in them having to disappoint these patients. Planners particularly dislike informing patients about the postponement of their surgeries when this has happened before. They “prefer to postpone a longer surgery, because then we have to disappoint only 1 patient, instead of 4” (ID4, physician–planner). A strategy to avoid disappointing patients is delaying informing patients about their scheduled surgery until a week in advance. This allows for last-minute changes without patients noticing, thereby avoiding potential distress or anger. Some planners admit dreading patients’ reactions. One planner explained that if a patient “is crying on the phone, I will try to reschedule the surgery as soon as possible. If a patient reacts that it is okay, then I am inclined to think okay, fine” (ID8, planner). Another planner mentioned that she tries to refrain from making decisions based on patients’ emotions, especially when they express anger.

The planner notices an error in the schedule for the following day, necessitating the postponement of one patient’s surgery. The planner selects a patient based on the duration of the surgery and confirms this with the physician before calling the patient. The planner notes: “they are not going to be happy.” To the patient, the planner explains that an emergency surgery has come up, looking at me with a guilty expression. (Observation 2, 13-03-2025)

### Professional Habits and Customs

Professional habits and customs affect the prioritization of patients awaiting elective surgery. Physicians’ views on using urgency scores reflect their preference for professional autonomy over standardized decision-making tools. While urgency scores are deemed helpful for dealing with scarcity during crises, such as the recent COVID-19 pandemic, physicians question their added value in routine care. Rather, they believe that urgency scores fail to capture the individual needs of patients. As one physician stated, these scores are “far too rough, far too simplistic” (ID5, physician). At the same time, physicians acknowledge that prioritization is “somewhat relative” (ID1, physician) and ‘a bit of a fuzzy process” (ID5, physician). While physicians recognized the risk of inconsistency and variation in decision making between patients, they attributed this to logistical constraints, such as dependency on other departments, limited postoperative beds, and unforeseen events. Even though physicians considered the (not) using of urgency scores imperfect, they resist using even more standardized decision-making tools: “dialogue within the department could be improved to level out how we determine urgency scores. But at the same time, I am not fond of formulating too many rules” (ID6, physician).

Furthermore, professional customs shape physicians’ expectations of health care provision and waiting times. Restricting or delaying access to care conflicts with physicians’ perceived role as patient advocates. According to one physician, “waiting lists should not exist in health care” (ID2, physician). Especially rescheduling surgeries on short notice is perceived as “really poor care,” because “we have sufficient resources to neatly plan this in advance. It all boils down to a lack of capacity of ORs and personnel. But, money, in the end, it is all about money” (ID5, physician). Physicians were unconvinced that they had any influence on this. Some departments experimented with keeping 1 OR time slot open every week to timely accommodate postponed patients. However, efficiency and maximization of OR capacity ultimately took precedence. Notably, all physicians noted that other departments faced fewer difficulties with planning, because they were believed to have fewer urgent patients or perform fewer multidisciplinary surgeries.

## Discussion

The aim of this study was to provide insight into the prioritization of patients awaiting elective surgery in an academic hospital in the Netherlands, including the assignment of urgency scores, and to explore explanations for discrepancies between assigned scores and actual waiting times. Despite a modest sample size, the combination of interviews and observations provided unique in-depth insight into decision making on the patient level by physicians and planners.

Regarding the prioritization of patients, our findings demonstrate that the process from assigning urgency scores to scheduling a surgery is complex and dynamic, even when using decision-making tools to guide and standardize decisions. Our findings suggest that physicians and planners hold different perceptions of the use of urgency scores. Physicians reluctantly accept the use of urgency scores and consider them inadequate for capturing the complexity of patients’ needs compared with their own clinical judgment. In contrast, planners rely on urgency scores as a primary indicator of clinical priority when scheduling surgeries. While physicians may regard the planners’ professional autonomy as being limited in this context, our findings suggest it is actually pivotal in decision-making practice. Much of their work may remain invisible, but planners demonstrate expert skills—coordinating different tasks and navigating between the individual and the many under continuously changing circumstances—that are essential for the efficient and safe provision of care.

Our findings also suggest that urgency scores assigned by physicians may not always reflect the “true” clinical priority, as these may be influenced by interactions with the patient and used strategically to give their patients an advantage. Similarly, planners’ decisions on the prioritization of patients are shaped, perhaps unconsciously, by their perceptions of patients. These findings are consistent with previous research suggesting that nonclinical, subjective factors play a considerable role in clinical decision making.^9–11,21^ Nonetheless, further research is necessary to examine whether in the context of the current study such actions are implicitly biased by nonmedical factors or instead stem from internalized (medical) expertise.

With regard to discrepancies between assigned urgency scores and actual waiting times in the hospital, our findings suggest that discrepancies may arise from the extensive professional autonomy, combined with the role of emotions in decision-making practice. It was evident that physicians and planners are committed to the health and well-being of patients and delivering the highest possible quality of care. Yet, decisions made to schedule surgery for one patient may inadvertently result in longer waiting times for others. Moreover, discrepancies may arise from logistical constraints, particularly the duration and multidisciplinary nature of a surgery, and the availability of postop beds, that hinder strict adherence to prioritization based on clinical urgency. Our findings suggest that while longer and multidisciplinary procedures may be more challenging to schedule, once scheduled, they are less likely to be postponed.

In addition, it is notable that, even though inconsistencies in the assignment of urgency scores were noticed, prioritization decisions were rarely reviewed or discussed within the 4 departments. For example, physicians noticed that colleagues consistently assigned higher urgency scores to their patients. However, our findings also suggest that established traditions of professional autonomy and hierarchical dynamics rooted in medical expertise hamper physicians from challenging each other’s prioritization decisions. Facilitating regular review of the prioritization practices within the department may enhance consistency and reduce variation or at least enhance transparency in decision making.

The findings of other studies suggest that potential variation may disproportionately affect patients with lower socioeconomic status or those who face language barriers.^21^ As our study did not focus on any specific group of patients, we are unable to draw conclusions about whether variation in prioritization disproportionately affects patients with lower socioeconomic status or those who face language barriers. However, our findings do suggest that patients who (repeatedly) contact planners and actively express their frustration may be scheduled for surgery sooner than those who wait more passively for updates about their surgery. Further research is needed to examine whether observed aspects of the prioritization of patients awaiting elective surgery affect some patient groups more than others do and to what extent this is considered justifiable and desirable.

This study was conducted in an academic hospital in the Netherlands, and our findings may be regarded as being specific to this setting. Further research is needed to examine the extent to which our findings are generalizable to other settings (i.e., general hospitals). Nonetheless, we expect that similar challenges—financial, staffing and capacity shortages, and organizational structures—likely exist in other settings based on the correspondence between our findings and those of others.^9–11,21^

In conclusion, while greater standardization and a more consistent use of urgency scores may enhance fairness and transparency in prioritization, our findings indicate that these tools inadequately capture the complexity of patients’ needs, organizational requirements for scheduling surgeries, as well as the nonclinical factors that influence decision making. Moreover, physicians may accept such standardized approaches in hospital settings but not necessarily use them as consistently as intended. Importantly, effective prioritization depends on the situated expertise and tacit knowledge of physicians and planners. Rather than relying solely on stricter adherence to urgency scores, hospital leaders may strengthen prioritization processes by fostering communication and collaboration between physicians and planners through regular interdisciplinary meetings and systematic feedback exchanges. Such efforts can complement standardization and support a more integrated and context-sensitive approach that considers capacity constraints, differences between surgical specialties, daily fluctuations in caseload and staffing, and the specific needs of individual patients.

## Supplemental Material

sj-docx-1-mdm-10.1177_0272989X261422220 – Supplemental material for “They Are Not Going to Be Happy”: An Ethnographic Study of the Prioritization of Patients Awaiting Elective Surgery in an Academic Hospital in the Netherlands Supplemental material, sj-docx-1-mdm-10.1177_0272989X261422220 for “They Are Not Going to Be Happy”: An Ethnographic Study of the Prioritization of Patients Awaiting Elective Surgery in an Academic Hospital in the Netherlands by Philipa Mos, Bert de Graaff, Michelle Heijke, Hester Lingsma, Robert Jan Baatenburg de Jong and Vivian Reckers-Droog in Medical Decision Making

sj-docx-2-mdm-10.1177_0272989X261422220 – Supplemental material for “They Are Not Going to Be Happy”: An Ethnographic Study of the Prioritization of Patients Awaiting Elective Surgery in an Academic Hospital in the Netherlands Supplemental material, sj-docx-2-mdm-10.1177_0272989X261422220 for “They Are Not Going to Be Happy”: An Ethnographic Study of the Prioritization of Patients Awaiting Elective Surgery in an Academic Hospital in the Netherlands by Philipa Mos, Bert de Graaff, Michelle Heijke, Hester Lingsma, Robert Jan Baatenburg de Jong and Vivian Reckers-Droog in Medical Decision Making
