# Supplementary material for: “They Are Not Going to Be Happy”: An Ethnographic Study of the Prioritization of Patients Awaiting Elective Surgery in an Academic Hospital in the Netherlands
Source: Med Decis Making. 2026 Feb 21;46(4):480–8. doi: 10.1177/0272989X261422220 (PMC13062453; doi:10.1177/0272989X261422220)
Supplement: sj-docx-2-mdm-10.1177_0272989X261422220 – Supplemental material for “They Are Not Going to Be Happy”: An Ethnographic Study of the Prioritization of Patients Awaiting Elective Surgery in an Academic Hospital in the Netherlands [file sj-docx-2-mdm-10.1177_0272989X261422220.docx]

**SUPPLEMENTARY MATERIALS**

**Supplementary Material 1 – Topic list for the semi-structured interviews**

**Background & objectives**

Surgical capacity is scarce and, therefore, patients are frequently put on waiting lists for elective surgery.

In this study, researchers from the Erasmus Medical Centre and the Erasmus School of Health Policy & Management are aiming to get a better understanding of the decision-making process that results in the planning of patients for elective surgery. Thank you for your willingness to partake in this interview. With your permission, I plan to record the conversation. The audio recording will be converted to text. I will not share with the other researchers, nor will it be possible to trace your statements back to you, or your department, in the publication of our findings. Do you have any questions at this time about the study, or about the interview?

**Start of the interview**

*Introductory question:* Can you describe the process of scheduling patients for surgery, from the moment you determine that there is an indication for surgery, and what is your role in it?

***Urgency scores***

1. Assessment of urgency scores
   1. Actors involved in assessment of urgency scores
   2. Considerations in the assessment of urgency scores
   3. Individual or collective decision-making
2. Difficulties in assessing urgency scores
   1. Doubts/ uncertainty about what score is fitting
   2. Example of a situation where it is very clear what the urgency score is, and an example where it is less clear?
   3. What matters play a decisive role in the latter case?

***Planning of patients for surgery***

1. Actors involved in planning patients for surgery
   1. Roles and responsibilities
   2. Dynamics
   3. Communication

**Additional questions**

1. Advantages and disadvantages of the decision-making process
2. Consultation structure/systematics within the department/ between involved actors
3. Perceptions of hospital-wide surgical capacity; effect on assessment of urgency scores or prioritization of patients for surgery.
4. Comparisons with other surgical departments in assessment of determine urgency scores and prioritization.

**Conclusion**

*Final question*: Have we missed any matters that are relevant to the planning and prioritization of patients for elective surgery during this conversation?
